# The opioid effects of gluten exorphins: asymptomatic celiac disease

**DOI:** 10.1186/s41043-015-0032-y

**Published:** 2015-11-24

**Authors:** Leo Pruimboom, Karin de Punder

**Affiliations:** 1 Natura Foundation, Edisonstraat 66, 3281 NC Numansdorp, Netherlands; 2 Department of Laboratory Medicine, University Medical Center Groningen (UMCG), University of Groningen, P.O. Box 30.001, 9700 RB Groningen, Netherlands; 3 Institute of Medical Psychology, Charité University Medicine Berlin, Hufelandweg 14, 10117 Berlin, Germany

**Keywords:** Asymptomatic celiac disease, Celiac disease, Gliadin, Gluten, Exorphins

## Abstract

Gluten-containing cereals are a main food staple present in the daily human diet, including wheat, barley, and rye. Gluten intake is associated with the development of celiac disease (CD) and related disorders such as diabetes mellitus type I, depression, and schizophrenia. However, until now, there is no consent about the possible deleterious effects of gluten intake because of often failing symptoms even in persons with proven CD. Asymptomatic CD (ACD) is present in the majority of affected patients and is characterized by the absence of classical gluten-intolerance signs, such as diarrhea, bloating, and abdominal pain. Nevertheless, these individuals very often develop diseases that can be related with gluten intake. Gluten can be degraded into several morphine-like substances, named gluten exorphins. These compounds have proven opioid effects and could mask the deleterious effects of gluten protein on gastrointestinal lining and function. Here we describe a putative mechanism, explaining how gluten could “mask” its own toxicity by exorphins that are produced through gluten protein digestion.

## Background

Gluten is the main structural protein complex of wheat consisting of glutenins and gliadins. Glutenins are polymers of individual proteins and are the fraction of wheat proteins that are soluble in dilute acids. Prolamins are the alcohol-soluble proteins of cereal grains that are specifically named gliadins in wheat [1], which can be further degraded to a collection of opioid-like polypeptides called *exorphins* in the gastrointestinal tract [2]. Gliadin epitopes from wheat gluten and related prolamins from other gluten-containing cereal grains, including rye and barley, can trigger celiac disease (CD) in genetically susceptible people [3], and accumulating data provide evidence for the deleterious effects of gluten intake on general human health. Nevertheless, until now, there is no consent about the possible detrimental health effects of gluten intake because of often failing gastrointestinal symptoms even in individuals with proven CD. By describing our “silent opioid hypothesis,” we hope to shine light on this highly conflictive scientific item. Our review process and literature search was based on the use of the following key words: gluten, gliadin, celiac disease, asymptomatic celiac disease, gluten and transglutaminase, gluten and exorphins, gluten and intolerance, gluten and DPP IV, gluten and substance P, DPP IV, gluten and neoantigens, celiac disease and epidemiology, and gluten-free diet. Literature inclusion criteria included in vitro, in vivo, and human trial studies; indexed publications; full-text papers; and research methodology. Papers were excluded when not indexed and when methodology did not reach minimal criteria, and papers older than 2005 were excluded when more actual publications were available.

## Asymptomatic celiac disease

CD normally presents itself with a number of typical signs and symptoms of malabsorption: diarrhea, muscle wasting, and weight loss. Other gastrointestinal (GI) symptoms like abdominal pain, bloating, and flatulence are also common. Curiously, a large group of patients that have been diagnosed with CD through screening for CD-specific antibodies and duodenal biopsy [3, 4] lack these classical symptoms, a condition that is also referred to as “asymptomatic CD” (ACD). Many disorders are present in patients with ACD, including diabetes mellitus type I [5, 6], severe hypoglycemia in diabetes mellitus type I [7], psoriasis [8], sleep apnea in children [9], neoplasia [10], atopic dermatitis [11], depression [8], subclinical synovitis in children [12], autism [13], schizophrenia [14], and irritable bowel syndrome (IBS) [8], suggesting that gluten intake is related to the development of these conditions.

ACD is present in a large group of diagnosed celiac patients [15, 16]. A study based on the data of the National Health and Nutrition Examination Survey showed that only 17 % of patients with serologically diagnosed CD suffer from the classical celiac symptoms [17]. A human study in 2089 elderly individuals looking for possible persistence of anti-gliadin antibody (AGA) positivity showed that 54 % of the AGA-positive patients suffered from intestinal inflammation, but only a small number of them complained about gastrointestinal symptoms [18]. The rate of elderly people suffering from mild inflammation in the gut mucosa and being AGA-negative is, according to a recent Swedish-population-based study, only 3.8 % [19], again showing that gluten can cause inflammatory injury in the gut, without suffering any gastrointestinal symptoms. The presence of possible ACD is further recognized by the National Institute for Health and Care Excellence (UK) [20]. According to the guidance for CD screening issued in 2009, it is recommended to screen for CD when patients suffer from diabetes mellitus type I, IBS, thyroid hormone disturbances, Addison’s disease, epilepsy, lymphoma, rickets, repetitive miscarriage, Sjögren’s disease, and Turner disease. The following question arises: why do patients with ACD, with proven inflammatory signs, not suffer from pain, bloating, and other typical symptoms? Could it be that substances present in gluten with opioid effects mask the deleterious effects, functioning as masking compounds of gastrointestinal symptoms, converting the causal factor of CD, gluten, into a silent killer?

## CD is characterized by the presence of serum antibodies against tissue transglutaminase

The most reliable way to diagnose CD is through small intestinal biopsy and measurement of the presence of serum antibodies against tissue transglutaminase (tTG), the main endomysial auto-antigen in CD [21–23]. Tissue TG deamidates glutamine residues from the gliadin peptide into glutamic acid, leading to enhanced immunogenicity of the resulting modified peptides. In addition, tTG can, in the absence of any other protein substrate, crosslink with gliadin, producing a tTG-gliadin complex, which can be considered a neo-antigen with possible immune toxicity [24, 25]. Both symptomatic and asymptomatic CD are associated with certain immune system-related genetic polymorphisms, of which the HLA-DQ2 and HLA-DQ8 polymorphisms are expressed in the majority of CD patients [3]. However, many more genes, all related to a more pro-inflammatory activity of the immune system, are also linked with increased CD susceptibility [26]. A recent study by Sironi et al. [27] showed that several interleukin/interleukin receptor genes, involved in the pathogenesis of CD, have been subjected to pathogen-driven selective pressure. Particularly, CD alleles of IL18-RAP, IL18R1, IL23, IL18R1, and the intergenic region between IL2 and IL21 display higher frequencies in populations exposed to high microbial/viral loads, suggesting that these variants protected humans against pathogens. Since CD occurred after the increase of hygiene management and the incorporation of cereals into the human diet, it can be assumed that individuals bearing these genotypes are better protected against pathogens but at the same time are more susceptible for autoimmune diseases in general and CD specifically [28]. Although the above-described events explain the development of the typical inflammatory symptoms of CD and even the flattening of the gut lining through this immune response, they do not explain the phenomenon that many patients suffer from ACD at the level of the intestine and often, in parallel, suffer from extra-gastrointestinal disorders [8].

## Gliadin is degraded to a collection of polypeptides called exorphins in the gastrointestinal tract

Breakdown of gliadin from wheat is achieved through hydrolysation by intestinal pepsin, leucine aminopeptidase, and elastase, resulting in the release of immune-reactive and opioid-like peptides, including gliadinomorphin-7 (Tyr-Pro-Gln-Pro-Gln-Pro-Phe) from α-gliadin [2]. Further breakdown of these peptides, which are rich in proline, depends on the enzyme dipeptidyl peptidase IV (DPP IV), capable of cleaving N-terminal dipeptides with proline at the second (penultimate) position [29–31]. The remaining tripeptide (in the case of gliadinomorphin-7) with proline in the center is slowly hydrolyzed and acts as a selective competitive inhibitor for DPP IV [32–34].

Whereas total breakdown of gliadin into isolated amino acids prevents the presence of the gluten epitopes which are known to provoke a pro-inflammatory response of the immune system in genetically susceptible people [35, 36], a possible DPP IV deficiency/inactivity could result in the incomplete breakdown of gluten, and thereby increase the presence of immune-reactive and opioid-like peptides, also known as gluten exorphins [36–39]. Gluten is not the only source of exorphins. Dairy products and certain vegetables such as soy and spinach also contain proteins, which can be converted in bioactive exorphins [40].

**Fig. 1:**
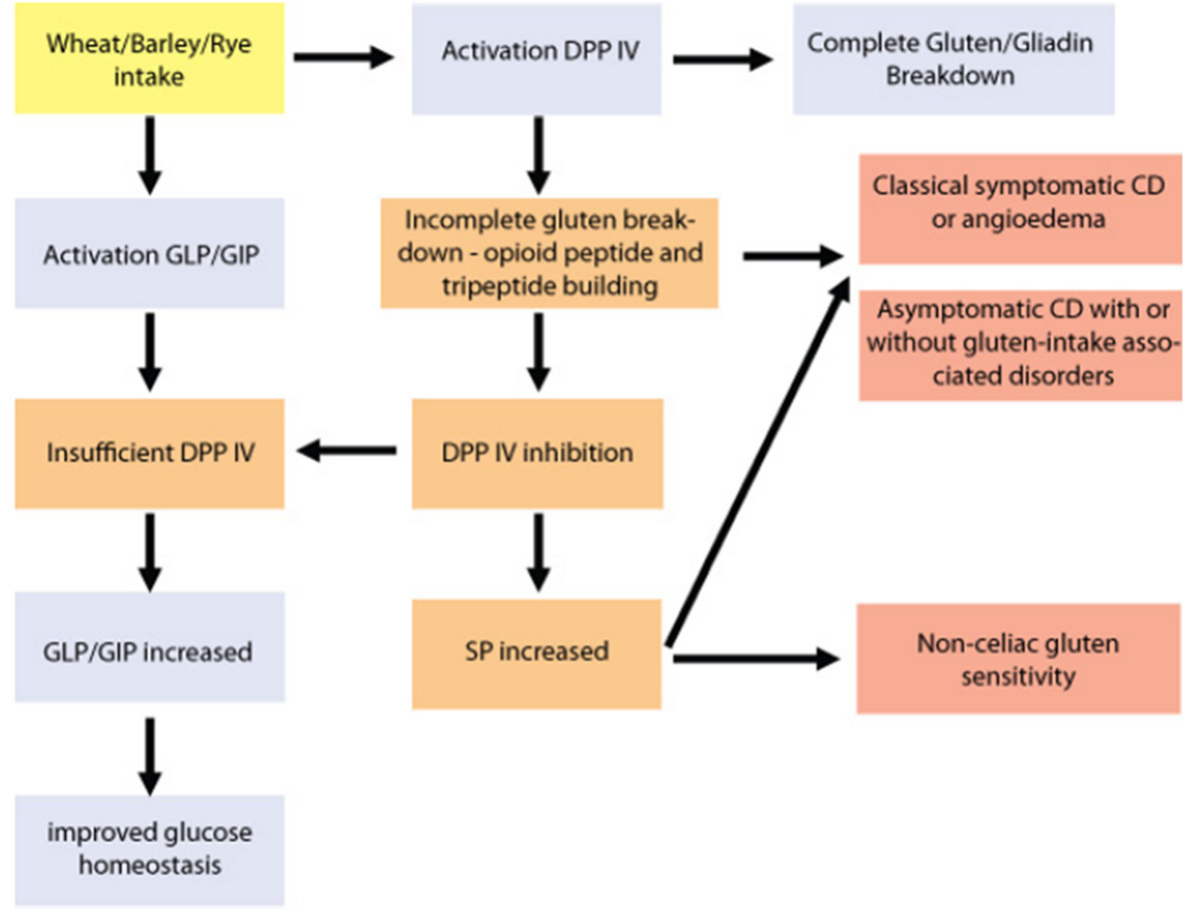
The development of symptomatic and asymptomatic CD and NCGS. Incomplete gluten breakdown results in inhibition of DPP IV and the possible increase of SP, leading to intestinal and extra-intestinal gluten-induced disorders. Gluten-derived DPP IV inhibition also increases the presence of GIP and GLP in the gut, leading to improved glucose homeostasis

Gliadin from gluten and casein from dairy products show surprisingly high substrate specificity for DPP IV when compared with other endogenous DPP IV substrates. For example, DPP IV shows higher affinity for gliadin and casein than for substance P (SP) [41] and glucagon-like peptide (GLP) [42]. Gliadin is highly specific for DPP IV [36], which is further evidenced by its binding affinity with human DPP IV. By using an enzyme-linked immunosorbent assay, it was shown that binding of gliadin and casein to DPP IV inhibited DPP IV binding to anti-DPP IV by 52 and 44 %, respectively [43]. The fact that gliadin has a high affinity for DPP IV might explain why so many patients with proven CD are asymptomatic. Inhibition of DPP IV by gliadin can result in increased levels of non-metabolized gliadin molecules with opioid activity that can inhibit the typical abdominal pain associated with classical CD (Fig. 1).

## Opioid pathways could be responsible for the development of ACD

It is surprising that a large group of patients, positive for the presence of CD antibodies and with proven histological CD, do not suffer from any gastrointestinal symptoms. If it is the opioid effects of gluten itself masking the classical symptoms of CD, then symptoms should be provoked when patients are given naloxone, a natural antagonist of morphine.

### Opioid effects on intestinal transit time

Gastric emptying and intestinal transit are influenced by endogenous and exogenous opioid substances. It is known for long that morphine increases gastrointestinal transit time in humans and that this can be reversed by naloxone [44]. Early research showed that gluten exorphins induced a significant increase in transit time, and this effect was abolished when naloxone was administered [45]. A more recent study supports these early findings. In a single-center study, Urgesi et al. [46] observed that patients suffering from CD show a significantly longer small bowel transit time. In the discussion, the authors mention different pathways explaining their findings but do not mention the possible effect of opioids on intestinal transit time.

### Gluten-derived exorphins mimic endogenous opioid activity

Stimulation of insulin production after meal intake is considered an endogenous opioid activity. Early research in rodents showed that oral administration of gluten exorphin A5 stimulated insulin production after food intake. The postprandial increase of insulin release by gluten exorphin was completely abolished by the opioid antagonist naloxone, implying that gluten exorphins maintain bioavailability for the peripheral nervous system within the gastrointestinal tract and pancreatic tissues [47]. Elevated circulating prolactin levels were observed in individuals diagnosed with CD [48]. A short gluten-free diet period lowered prolactin levels in these patients, suggesting that gluten (or gluten-derived substances), similar to endogenous opioids, directly affects prolactin secretion. This was further evidenced by Fanciulli et al. [49]. In rats, by using an opioid antagonist unable to cross the blood-brain barrier (naloxone methobromide), intracerebroventricular (ICV)-injected gluten exorphins stimulated prolactin release through activation of opioid receptors probably also outside the brain.

### Gluten exorphins influence behavior and pain perception

A recent review of the literature concluded that food-derived exorphins are bioactive and affect behavioral traits such as spontaneous behavior, memory, and pain perception in rodents. The highest behavioral influence was measured for casein and spinach-derived exorphins (respectively, B-casomorphin and rubiscolin) [50]. Only one of the reviewed studies described the effects of gliadin exorphins in this context. Takahashi et al. [51] showed that ICV-administered gliadin exorphin A5 induced antinociceptive effects and orally delivered gliadin exorphin A5 modified learning and anxiety behavior during several laboratory stressors in mice, thus indicating that orally delivered exorphins can influence both the peripheral and central nervous system and suggesting that gluten exorphins possess opioid activity that could potentially mask symptoms in ACD patients.

Besides explaining the lack of intestinal symptoms through gluten exorphin opioid activity in individuals suffering from ACD, DPP IV inhibition by gluten intake can have many other consequences on human health. DPP IV inhibition is known to have anti-diabetic effects but at the same time could be responsible for the presence of extra-intestinal symptoms and disorders in ACD and the occurrence of intestinal and extra-intestinal symptoms and disorders in CD and non-celiac gluten sensitivity (NCGS) patients (described below).

## DPP IV inhibition by gluten intake

### DPP IV blockage by gliadin peptides improves glucose homeostasis

Casein is not the only protein competing with gluten as a substrate for DPP IV (a nice overview of natural DPP IV substrates is provided by Gorrel et al. [52]). Other N-terminal dipeptides with proline at the second position, like the incretins, GLP and glucose-dependent insulinotropic polypeptide (GIP), both important regulators of glucose metabolism and essential to gut function, compete with gluten as substrates for DPP IV [52–57]. Carbohydrate intake increases the secretion of both incretins, which are normally rapidly broken down by DPP IV [58]. In a recent review [58], it is described how the inhibiting effect of gliadin on DPP IV increases the presence of GLP and GIP, by using whole wheat as a natural DPP IV inhibitor. DPP IV inhibition by gliadin could explain the “health promoting” effects of whole wheat intake, as the suppression of GLP and GIP breakdown has anti-diabetic effects [58] (Fig. 1). Contrasting data were found in a randomized, controlled, and open-labeled study [59]. Two days of DPP IV inhibition increased GLP and GIP levels but did not affect glucose values, transit time, or gastric emptying in healthy subjects, suggesting that short exposure to DPP IV inhibition does not affect any function related with DPP IV. The latter makes sense when observing the toxic effects of gluten as a natural DPP IV inhibitor. When patients suffering from NCGS followed a gluten-free diet and were re-challenged with gluten intake, it took approximately 7 days before new symptoms were provoked [60]. Longer use of gluten and synthetic DPP IV inhibitors have been shown to influence gastric emptying and transit time significantly. It is even so that deceleration and slowing of transit time are considered the most important mechanisms by which DPP IV inhibitors influence glucose homeostasis [56, 61, 62].

### DPP IV blockage by gliadin peptides induces intestinal and extra-intestinal disorders

Breakdown of SP is also dependent on DPP IV activity. SP has neurological, immunological, and endocrinological functions and influences pain sensitivity, gut peristaltic, inflammation, and social interaction [63]. Increased concentrations of SP in the gut can produce abdominal pain with diarrhea [64] and angioedema with swelling and abdominal pain [65]. High SP levels in the gut can even produce pancreatitis together with abdominal pain, diarrhea, and vomiting [66]. A recent double-blind placebo-controlled human trial showed that the intake of a small amount of gluten (4.375 g/day for 1 week) significantly increased intestinal and extra-intestinal symptoms in individuals with self-reported gluten sensitivity [67]. Typical intestinal symptoms such as bloating and abdominal pain were increased after 1 week of gluten intake, as were extra-intestinal symptoms, such as depression and apthous stomatitis. Because in patients suffering from NCGS no known intestinal lesions or other biomarkers such as antibodies against gluten/gliadin/self-antigens seem to be present [68], their symptoms have to be explained by different pathways. NCGS presents itself with intestinal symptoms like diarrhea, abdominal discomfort, and flatulence, while headache, lethargy, attention-deficit/hyperactivity disorder, ataxia, or oral ulceration appears as an extra-intestinal symptom [3, 67]. Most, if not all, of these symptoms can be explained by increased levels of SP [64, 69, 70], suggesting that, in NCGS patients, gliadin blocks DPP IV activity and thereby inhibits SP breakdown. Thus, interventions targeting SP release in this group of patients could be a possible strategy to alleviate their intestinal and extra-intestinal symptoms [67] (Fig. 1).

### DPP IV inhibition increases the development of angioedema

DPP IV inhibition by synthetic inhibitors, such as sitagliptin, is known to increase the possibility of developing angioedema [71]. CD produces the same symptoms as angioedema, and both disorders are so similar that, in general, it is advised to screen people with hereditary angioedema for CD [72]. Skin disorders, CD, and angioedema seem to be associated as seen in patients suffering from gluten-induced chronic urticaria [73] of which approximately 40 % also experience angioedema [74]. Even guidelines for the management of urticaria are similar as for angioedema [74, 75], suggesting that both disorders have the same etiology, which could be CD and/or increased levels of SP through DPP IV inhibition. A study by Ramsay et al. [76] indicated that patients with gastrointestinal disorders (CD, morbus Crohn, colitis) suffer from mast-cell-induced inflammation. Interestingly enough, mast-cell inflammation can be induced by SP [77]. DPP IV inhibition by gluten would also explain the relationship between gluten intake and skin disorders [78, 79]. Many skin diseases, including acne vulgaris, are associated with higher serum levels of SP [69], which can be induced by blockage of DPP IV [65]. Another gluten intake-related disorder, major depression, is also related with DPP IV inhibition; low serum DPP IV is an important marker for depression [80], and gluten could function as an inhibitor of DPP IV.

### Gliadin peptides can cause anatomical changes at the level of the brain

Recent research in humans has shown that gluten intake can even cause anatomical changes at brain level, although neurological symptoms are absent. Anatomical MRI shows silent neurological changes, including bilateral decrease in cortical gray matter and caudate nuclei volumes in celiac patients compared to controls [81]. Negative correlations were found between the duration of the disease and the volumes of the affected regions. Similar neurological changes were observed in a retrospective examination of the brain by MRI of patients suffering from biopsy-proven CD who were referred for a neurological opinion by their gastroenterologist [82]. Patients were divided into subgroups based on their primary neurological complaint (balance disturbance, headache, and sensory loss). The outcome was that CD patients suffer from a significant loss of cerebellar volume compared with healthy controls. Affected area were brain regions below and above the tentorium cerebelli. These changes were the highest in the headache subgroup and unexpected for the patient’s age. The headache group had an average loss of white matter in these regions two times more than the subgroup with balance disturbance and six times more than the subgroup suffering from sensory loss. One possible explanation for the loss of white matter in people suffering from CD is the presence of gluten-induced autoimmune vasculitis [83]. Another more recent hypothesis explaining the loss of white matter caused by reactions against gluten is related with the complementary immune system. The complement protein C1Q is known to bind and help eliminate complexes of immune globulins bound to antigens [84]. C1Q further is considered a “punishment factor” of the central neurological system, produced by strong synapsis and marking weak synapsis for possible phagocytosis by neighboring glia cells [85]. The process of synapsis breakdown should be considered normal in early life with the purpose of remodeling the central nervous system during neurological development [86]. Increased expression of C1Q has been observed in patients suffering from Alzheimer [87], autism [88], and schizophrenia [89]. Severance et al. [84] showed that C1Q binds preferentially to immune globulins coupled with casein and gluten antigens. Their results suggest that the increased expression of C1Q increased synaptic breakdown and could be responsible for schizophrenia onset. We speculate that the increased presence of gliadin peptides, induced by DPP IV inhibition, could be responsible for stimulating C1Q expression and, thereby increases disease susceptibility for these neurodevelopmental and neurodegenerative disorders.

## Conclusions

The precise pathway leading to the development of ACD still needs to be discovered. However, the putative mechanism presented in this review could explain this intruding phenomenon. The incomplete breakdown of the gluten protein, resulting in the presence of gliadin peptides with opioid effects, makes it plausible to suggest that the opioid effects of gluten exorphins could be responsible for the absence of classical gastrointestinal symptoms of individuals suffering from gluten-intake-associated diseases. Moreover, the partial digestion of gluten, leading to DPP IV inhibition, could also account for the presence of extra-intestinal symptoms and disorders in ACD and the occurrence of intestinal and extra-intestinal symptoms and disorders in CD and NCGS patients. If so, then individuals suffering from any of these conditions should be recognized in time and engage in a gluten-free lifestyle to prevent gluten-induced symptoms and disorders.

## References

[1] Tatham AS, Shewry PR (2008). Allergens to wheat and related cereals. Clin Exp Allergy.

[2] Trivedi MS, Shah JS, Al-Mughairy S, Hodgson NW, Simms B, Trooskens GA (2014). Food-derived opioid peptides inhibit cysteine uptake with redox and epigenetic consequences. J Nutr Biochem.

[3] Troncone R, Jabri B (2011). Coeliac disease and gluten sensitivity. J Intern Med.

[4] Strohle A, Wolters M, Hahn A (2013). Celiac disease--the chameleon among the food intolerances. Med Monatsschr Pharm.

[5] Bybrant MC, Ortqvist E, Lantz S, Grahnquist L (2014). High prevalence of celiac disease in Swedish children and adolescents with type 1 diabetes and the relation to the Swedish epidemic of celiac disease: a cohort study. Scand J Gastroenterol.

[6] Hansson T, Dahlbom I, Tuvemo T, Frisk G (2015). Silent coeliac disease is over-represented in children with type 1 diabetes and their siblings. Acta Paediatr.

[7] Khoury N, Semenkovich K, Arbelaez AM (2014). Coeliac disease presenting as severe hypoglycaemia in youth with type 1 diabetes. Diabet Med.

[8] Pinto-Sanchez MI, Bercik P, Verdu EF, Bai JC (2015). Extraintestinal manifestations of celiac disease. Dig Dis.

[9] Parisi P, Pietropaoli N, Ferretti A, Nenna R, Mastrogiorgio G, Del Pozzo M (2015). Role of the gluten-free diet on neurological-EEG findings and sleep disordered breathing in children with celiac disease. Seizure.

[10] Brito MD, Martins A, Henrique R, Mariz J (2014). Enteropathy-associated T cell lymphoma as a complication of silent celiac disease. Hematol Rep.

[11] Ress K, Annus T, Putnik U, Luts K, Uibo R, Uibo O (2014). Celiac disease in children with atopic dermatitis. Pediatr Dermatol.

[12] Iagnocco A, Ceccarelli F, Mennini M, Rutigliano IM, Perricone C, Nenna R (2014). Subclinical synovitis detected by ultrasound in children affected by coeliac disease: a frequent manifestation improved by a gluten-free diet. Clin Exp Rheumatol.

[13] Knivsberg AM, Reichelt KL, Hoien T, Nodland M (2002). A randomised, controlled study of dietary intervention in autistic syndromes. Nutr Neurosci.

[14] Jackson J, Eaton W, Cascella N, Fasano A, Warfel D, Feldman S (2012). A gluten-free diet in people with schizophrenia and anti-tissue transglutaminase or anti-gliadin antibodies. Schizophr Res.

[15] Anderson RP, Henry MJ, Taylor R, Duncan EL, Danoy P, Costa MJ (2013). A novel serogenetic approach determines the community prevalence of celiac disease and informs improved diagnostic pathways. BMC Med.

[16] Bizzaro N, Tozzoli R, Villalta D, Fabris M, Tonutti E (2012). Cutting-edge issues in celiac disease and in gluten intolerance. Clin Rev Allergy Immunol.

[17] Rubio-Tapia A, Ludvigsson JF, Brantner TL, Murray JA, Everhart JE (2012). The prevalence of celiac disease in the United States. Am J Gastroenterol.

[18] Ruuskanen A, Kaukinen K, Collin P, Krekela I, Patrikainen H, Tillonen J (2013). Gliadin antibodies in older population and neurological and psychiatric disorders. Acta Neurol Scand.

[19] Walker MM, Murray JA, Ronkainen J, Aro P, Storskrubb T, D’Amato M (2010). Detection of celiac disease and lymphocytic enteropathy by parallel serology and histopathology in a population-based study. Gastroenterology.

[20] National Institute for Health and Care Excellence. http://www.nice.org.uk/guidance/CG86/informationforpublic. Accessed May 2015. 25756143

[21] Dieterich W, Ehnis T, Bauer M, Donner P, Volta U, Riecken EO (1997). Identification of tissue transglutaminase as the autoantigen of celiac disease. Nat Med.

[22] Frulio G, Polimeno A, Palmieri D, Fumi M, Auricchio R, Piccolo E (2015). Evaluating diagnostic accuracy of anti-tissue Transglutaminase IgA antibodies as first screening for Celiac Disease in very young children. Clin Chim Acta.

[23] Fasano A, Catassi C (2012). Clinical practice, Celiac disease. N Engl J Med.

[24] Colomba MS, Gregorini A (2012). Are ancient durum wheats less toxic to celiac patients? A study of alpha-gliadin from Graziella Ra and Kamut. ScientificWorldJournal.

[25] Di Pisa M, Pascarella S, Scrima M, Sabatino G, Real-Fernandez F, Chelli M (2015). Synthetic peptides reproducing tissue transglutaminase-gliadin complex neo-epitopes as probes for antibody detection in celiac disease patients’ sera. J Med Chem.

[26] Qiao SW, Iversen R, Raki M, Sollid LM (2012). The adaptive immune response in celiac disease. Semin Immunopathol.

[27] Sironi M, Clerici M (2010). The hygiene hypothesis: an evolutionary perspective. Microbes Infect.

[28] Pruimboom L, Fox T, Muskiet FA (2014). Lactase persistence and augmented salivary alpha-amylase gene copy numbers might have been selected by the combined toxic effects of gluten and (food born) pathogens. Med Hypotheses.

[29] Augustyns K, Van der Veken P, Senten K, Haemers A (2005). The therapeutic potential of inhibitors of dipeptidyl peptidase IV (DPP IV) and related proline-specific dipeptidyl aminopeptidases. Curr Med Chem.

[30] De Meester I, Durinx C, Bal G, Proost P, Struyf S, Goossens F (2000). Natural substrates of dipeptidyl peptidase IV. Adv Exp Med Biol.

[31] Vanhoof G, Goossens F, De Meester I, Hendriks D, Scharpe S (1995). Proline motifs in peptides and their biological processing. FASEB J.

[32] Augustyns K, Bal G, Thonus G, Belyaev A, Zhang XM, Bollaert W (1999). The unique properties of dipeptidyl-peptidase IV (DPP IV / CD26) and the therapeutic potential of DPP IV inhibitors. Curr Med Chem.

[33] Mentlein R (1999). Dipeptidyl-peptidase IV, (CD26)--role in the inactivation of regulatory peptides. Regul Pept.

[34] Rahfeld J, Schierhorn M, Hartrodt B, Neubert K, Heins J (1991). Are diprotin A (Ile-Pro-Ile) and diprotin B (Val-Pro-Leu) inhibitors or substrates of dipeptidyl peptidase IV?. Biochim Biophys Acta.

[35] Bethune MT, Khosla C (2012). Oral enzyme therapy for celiac sprue. Methods Enzymol.

[36] Hausch F, Shan L, Santiago NA, Gray GM, Khosla C (2002). Intestinal digestive resistance of immunodominant gliadin peptides. Am J Physiol Gastrointest Liver Physiol.

[37] Detel D, Persic M, Varljen J (2007). Serum and intestinal dipeptidyl peptidase IV (DPP IV/CD26) activity in children with celiac disease. J Pediatr Gastroenterol Nutr.

[38] Ozuna CV, Iehisa JC, Gimenez MJ, Alvarez JB, Sousa C, Barro F (2015). Diversification of the celiac disease alpha-gliadin complex in wheat: a 33-mer peptide with six overlapping epitopes, evolved following polyploidization. Plant J.

[39] Shan L, Molberg O, Parrot I, Hausch F, Filiz F, Gray GM (2002). Structural basis for gluten intolerance in celiac sprue. Science.

[40] Teschemacher H (2003). Opioid receptor ligands derived from food proteins. Curr Pharm Des.

[41] Kikuchi M, Fukuyama K, Epstein WL (1988). Soluble dipeptidyl peptidase IV from terminal differentiated rat epidermal cells: purification and its activity on synthetic and natural peptides. Arch Biochem Biophys.

[42] Castillo GM, Reichstetter S, Bolotin EM (2012). Extending residence time and stability of peptides by protected graft copolymer (PGC) excipient: GLP-1 example. Pharm Res.

[43] Vojdani A (2005). Identification of etiology of autism.

[44] Yuan CS (1996). Gastric effects of mu-, delta- and kappa-opioid receptor agonists on brainstem unitary responses in the neonatal rat. Eur J Pharmacol.

[45] Tovoli F, Masi C, Guidetti E, Negrini G, Paterini P, Bolondi L (2015). Clinical and diagnostic aspects of gluten related disorders. World J Clin Cases.

[46] Urgesi R, Cianci R, Bizzotto A, Costamagna G, Riccioni ME (2013). Evaluation of gastric and small bowel transit times in coeliac disease with the small bowel PillCam(R): a single centre study in a non gluten-free diet adult Italian population with coeliac disease. Eur Rev Med Pharmacol Sci.

[47] Fukudome S, Shimatsu A, Suganuma H, Yoshikawa M (1995). Effect of gluten exorphins A5 and B5 on the postprandial plasma insulin level in conscious rats. Life Sci.

[48] Delvecchio M, Faienza MF, Lonero A, Rutigliano V, Francavilla R, Cavallo L (2014). Prolactin may be increased in newly diagnosed celiac children and adolescents and decreases after 6 months of gluten-free diet. Horm Res Paediatr.

[49] Fanciulli G, Dettori A, Tomasi PA, Demontis MP, Gianorso S, Anania V (2002). Prolactin and growth hormone response to intracerebroventricular administration of the food opioid peptide gluten exorphin B5 in rats. Life Sci.

[50] Lister J, Fletcher PJ, Nobrega JN, Remington G. Behavioral effects of food-derived opioid-like peptides in rodents: Implications for schizophrenia? Pharmacol Biochem Behav. 2015. doi:10.1016/j.pbb.2015.01.020. 10.1016/j.pbb.2015.01.020 25661529

[51] Takahashi M, Fukunaga H, Kaneto H, Fukudome S, Yoshikawa M (2000). Behavioral and pharmacological studies on gluten exorphin A5, a newly isolated bioactive food protein fragment, in mice. Jpn J Pharmacol.

[52] Gorrell MD (2005). Dipeptidyl peptidase IV and related enzymes in cell biology and liver disorders. Clin Sci (Lond).

[53] Aertgeerts K, Ye S, Shi L, Prasad SG, Witmer D, Chi E (2004). N-linked glycosylation of dipeptidyl peptidase IV (CD26): effects on enzyme activity, homodimer formation, and adenosine deaminase binding. Protein Sci.

[54] Blom WA, Lluch A, Stafleu A, Vinoy S, Holst JJ, Schaafsma G (2006). Effect of a high-protein breakfast on the postprandial ghrelin response. Am J Clin Nutr.

[55] Kim NH, Yu T, Lee DH (2014). The nonglycemic actions of dipeptidyl peptidase-4 inhibitors. Biomed Res Int.

[56] Tambascia MA, Malerbi DA, Eliaschewitz FG (2014). Influence of gastric emptying on the control of postprandial glycemia: physiology and therapeutic implications. Einstein (Sao Paulo).

[57] Tolle S (2014). GLP-1 analogues in treatment of type 1 diabetes mellitus. Dtsch Med Wochenschr.

[58] Cavazos A (2013). Mejia EGd. Identification of bioactive peptides from cereal storage proteins and their potential role in prevention of chronic diseases. Comprehensive Reviews in Food Science and Food Safety.

[59] Rhee NA, Ostoft SH, Holst JJ, Deacon CF, Vilsboll T, Knop FK (2014). The impact of dipeptidyl peptidase 4 inhibition on incretin effect, glucose tolerance, and gastrointestinal-mediated glucose disposal in healthy subjects. Eur J Endocrinol.

[60] Biesiekierski JR, Newnham ED, Irving PM, Barrett JS, Haines M, Doecke JD (2011). Gluten causes gastrointestinal symptoms in subjects without celiac disease: a double-blind randomized placebo-controlled trial. Am J Gastroenterol.

[61] Edholm T, Degerblad M, Gryback P, Hilsted L, Holst JJ, Jacobsson H (2010). Differential incretin effects of GIP and GLP-1 on gastric emptying, appetite, and insulin-glucose homeostasis. Neurogastroenterol Motil.

[62] Tasyurek HM, Altunbas HA, Balci MK, Sanlioglu S (2014). Incretins: their physiology and application in the treatment of diabetes mellitus. Diabetes Metab Res Rev.

[63] Karl T, Hoffmann T, Pabst R, von Horsten S (2003). Extreme reduction of dipeptidyl peptidase IV activity in F344 rat substrains is associated with various behavioral differences. Physiol Behav.

[64] Sohn W, Lee OY, Lee SP, Lee KN, Jun DW, Lee HL (2014). Mast cell number, substance P and vasoactive intestinal peptide in irritable bowel syndrome with diarrhea. Scand J Gastroenterol.

[65] Grouzmann E, Livio F, Buclin T (2009). Angiotensin-converting enzyme and dipeptidyl peptidase IV inhibitors: an increased risk of angioedema. Hypertension.

[66] Waeber B, Buclin T, Grouzmann E (2010). Angioedema during ACE and DPP-4 inhibition. Rev Med Suisse.

[67] Di Sabatino A, Volta U, Salvatore C, Biancheri P, Caio G, De Giorgio R (2015). Small amounts of gluten in subjects with suspected nonceliac gluten sensitivity: a randomized, double-blind, placebo-controlled, cross-over trial. Clin Gastroenterol Hepatol.

[68] Di Sabatino A, Corazza GR (2012). Nonceliac gluten sensitivity: sense or sensibility?. Ann Intern Med.

[69] Bowe WP, Logan AC (2011). Acne vulgaris, probiotics and the gut-brain-skin axis - back to the future?. Gut Pathog.

[70] Herpfer I, Katzev M, Feige B, Fiebich BL, Voderholzer U, Lieb K (2007). Effects of substance P on memory and mood in healthy male subjects. Hum Psychopharmacol.

[71] Gosmanov AR, Fontenot EC (2012). Sitagliptin-associated angioedema. Diabetes Care.

[72] Csuka D, Kelemen Z, Czaller I, Molnar K, Fust G, Varga L (2011). Association of celiac disease and hereditary angioedema due to C1-inhibitor deficiency. Screening patients with hereditary angioedema for celiac disease: is it worth the effort?. Eur J Gastroenterol Hepatol.

[73] Caproni M, Bonciolini V, D’Errico A, Antiga E, Fabbri P (2012). Celiac disease and dermatologic manifestations: many skin clue to unfold gluten-sensitive enteropathy. Gastroenterol Res Pract.

[74] Powell RJ, Du Toit GL, Siddique N, Leech SC, Dixon TA, Clark AT (2007). BSACI guidelines for the management of chronic urticaria and angio-oedema. Clin Exp Allergy.

[75] Curto-Barredo L, Silvestre JF, Gimenez-Arnau AM (2014). Update on the treatment of chronic urticaria. Actas Dermosifiliogr.

[76] Ramsay DB, Stephen S, Borum M, Voltaggio L, Doman DB (2010). Mast cells in gastrointestinal disease. Gastroenterol Hepatol (N Y).

[77] Hide M, Yanase Y, Greaves MW (2007). Cutaneous mast cell receptors. Dermatol Clin.

[78] Abenavoli L, Proietti I, Leggio L, Ferrulli A, Vonghia L, Capizzi R (2006). Cutaneous manifestations in celiac disease. World J Gastroenterol.

[79] Kotze LM (2013). Dermatitis herpetiformis, the celiac disease of the skin!. Arq Gastroenterol.

[80] Maes M, De Meester I, Verkerk R, De Medts P, Wauters A, Vanhoof G (1997). Lower serum dipeptidyl peptidase IV activity in treatment resistant major depression: relationships with immune-inflammatory markers. Psychoneuroendocrinology.

[81] Bilgic B, Aygun D, Arslan AB, Bayram A, Akyuz F, Sencer S (2013). Silent neurological involvement in biopsy-defined coeliac patients. Neurol Sci.

[82] Currie S, Hadjivassiliou M, Clark MJ, Sanders DS, Wilkinson ID, Griffiths PD (2012). Should we be ‘nervous’ about coeliac disease? Brain abnormalities in patients with coeliac disease referred for neurological opinion. J Neurol Neurosurg Psychiatry.

[83] Pratesi R, Gandolfi L, Friedman H, Farage L, de Castro CA, Catassi C (1998). Serum IgA antibodies from patients with coeliac disease react strongly with human brain blood-vessel structures. Scand J Gastroenterol.

[84] Severance EG, Gressitt KL, Halling M, Stallings CR, Origoni AE, Vaughan C (2012). Complement C1q formation of immune complexes with milk caseins and wheat glutens in schizophrenia. Neurobiol Dis.

[85] Fourgeaud L, Boulanger LM (2007). Synapse remodeling, compliments of the complement system. Cell.

[86] Stevens B, Allen NJ, Vazquez LE, Howell GR, Christopherson KS, Nouri N (2007). The classical complement cascade mediates CNS synapse elimination. Cell.

[87] Benoit ME, Tenner AJ (2011). Complement protein C1q-mediated neuroprotection is correlated with regulation of neuronal gene and microRNA expression. The Journal of neuroscience : the official journal of the Society for Neuroscience.

[88] Ashwood P, Wills S, Van de Water J (2006). The immune response in autism: a new frontier for autism research. J Leukoc Biol.

[89] Zakharyan R, Khoyetsyan A, Arakelyan A, Boyajyan A, Gevorgyan A, Stahelova A (2011). Association of C1QB gene polymorphism with schizophrenia in Armenian population. BMC medical genetics.

